# Institutional Training for Pharmacists

**Published:** 1920-05-15

**Authors:** 


### Institutional Training for Pharmacists.

THE RULE OF THE RETAIL SHOP.

When the new regulations for the qualifying examination of the Pharmaceutical Society of Great Britain were made, The Hospital noted that no provision was made for the time spent in the dispensary of an institution to count as part of the compulsory curriculum laid down. A deputation representing the injustice of these regulations to institutional pharmacists presented a case to the Council of the Pharmaceutical Society some time ago, and by a majority decision it was decided to adhere to the regulation requiring that a student must serve the compulsory curriculum period in a "retail" shop.

The Pharmaceutical Council has, however, accepted the report of a special Committee which has been considering the question anew, and it has now been decided to amend the regulations governing the qualifying examination, so that pupils may take their practical training in an institution under the direct supervision of a qualified pharmacist. It may be noted that training may be taken only at institutions specially "approved" by the Pharmaceutical Council.

Most of the larger hospitals and infirmaries have well equipped dispensaries for the manufacture of pharmaceutical preparations and the dispensing of these for in- and out-patients, and the training which it is possible to acquire at these institutions is admirable for the pupil who intends to qualify and adopt the career of an institutional pharmacist.

